# General practitioner attitudes towards prescribing aspirin to carriers of Lynch Syndrome: findings from a national survey

**DOI:** 10.1007/s10689-017-9986-9

**Published:** 2017-04-22

**Authors:** Samuel G. Smith, Robbie Foy, Jennifer McGowan, Lindsay C. Kobayashi, John Burn, Karen Brown, Lucy Side, Jack Cuzick

**Affiliations:** 1 Leeds Institute of Health Sciences, University of Leeds, Floor 10, Worsley Building, Leeds, LS2 9JT UK; 2 Wolfson Institute of Preventive Medicine, Queen Mary University of London, London, UK; 3 Institute of Epidemiology and Healthcare, University College London, London, UK; 4 Harvard T. H. Chan School of Public Health, Center for Population and Development Studies, Harvard University, Cambridge, MA USA; 5 Institute of Genetic Medicine, Newcastle University, Newcastle, UK; 6 Department of Cancer Studies, University of Leicester, Leicester, UK; 7 Institute for Women’s Health, University College London, London, UK

**Keywords:** Aspirin, Implementation, Lynch Syndrome, Preventive therapy, Chemoprevention, Prescribing

## Abstract

A dose non-inferiority study comparing 100 mg, 300 mg and 600 mg of aspirin for cancer prevention among Lynch Syndrome carriers is underway (Colorectal Adenoma/Carcinoma Prevention Programme trial 3, CaPP3). To guide implementation of the findings, we investigated general practitioner (GP) attitudes towards aspirin prescribing for Lynch Syndrome carriers. We surveyed 1007 UK GPs (9.6% response rate). Using a within-subjects design, GPs read a statement on harms and benefits of aspirin and indicated their willingness to prescribe aspirin at three doses (100 mg, 300 mg, 600 mg). Approximately two-thirds (70.8%) of GPs had heard of Lynch Syndrome or its associated names, and among those 46.7% were aware of the cancer preventive effects of aspirin among carriers. Two-thirds (68.1%) of GPs reported feeling comfortable discussing harms and benefits of aspirin with a Lynch Syndrome patient. Willingness to prescribe was 91.3% at 100 mg, and declined to 81.8% at 300 mg and 62.3% at 600 mg (*p* < 0.001). In multivariable analyses, willingness to prescribe (600 mg) was higher among GPs ≥50 years (OR 1.46, 95% CI 1.03–2.07), more experienced GPs (OR 1.50, 95% CI 1.10–2.04), GPs who were aware of the cancer preventive effects of aspirin (OR 1.58, 95% CI 1.20–2.09), and those who reported seeing a Lynch Syndrome patient in practice (OR 1.44, 95% CI 1.01–2.05, *p* = 0.045). GPs report limited awareness of Lynch Syndrome and the preventive effects of aspirin among carriers. To ensure the optimal dose identified in the CaPP3 trial is readily available to patients, prescribing guidance and strategies to educate GPs should be developed.

## Introduction

In the UK, colorectal cancer (CRC) affects over 41,000 people annually and more than 16,000 people die of the disease every year [1]. At a conservative estimate, 3% of CRC is attributable to Lynch Syndrome, equating to approximately 28,600 cases worldwide per annum [2]. Lynch Syndrome, also known as hereditary non-polyposis colorectal cancer (HNPCC), is defined in terms of having a germline mutation in a DNA mismatch repair gene, including MLH1, MSH2, MSH6, or PMS2 [3]. The syndrome is characterised by the development of a spectrum of cancers, primarily of the colorectum and endometrium at an unusually young age. Lynch Syndrome affects 1–3 in 1200 people in the UK, making it the most common hereditary CRC condition [4].

There is ongoing interest in the use of aspirin to prevent cancer among carriers of Lynch Syndrome. The Colorectal Adenoma/Carcinoma Prevention Programme’s second trial (CaPP2) is the only randomised controlled trial to compare aspirin (600 mg) against placebo among Lynch Syndrome carriers (n = 861) [5]. Intention-to-treat analysis failed to show a significant reduction in CRC incidence, but secondary analyses using both per protocol analysis and taking multiple primary events into account reached statistical significance. Participants completing 2 years of aspirin treatment experienced over a 50% reduction in CRC incidence [5]. There was also evidence suggesting reduced incidence of other cancers associated with the syndrome. Aspirin use is associated with an age-dependent increased risk of bleeding, particularly gastrointestinal bleeding [6]. The extent to which lower doses of aspirin reduce cancer incidence among this patient group is unknown. To identify the optimal dose for Lynch Syndrome carriers, the CaPP3 trial was developed. CaPP3 is a non-inferiority trial comparing doses of 100 mg, 300 mg and 600 mg among 2000 Lynch Syndrome carriers in the UK. Participants will take their allocated dose for 2 years, at which point the study will become open-label at the same dose. Results of the CaPP3 trial are expected in 2020.

No UK guidance exists on the management of Lynch Syndrome patients in clinical genetics, however the European Guidelines for the clinical management of Lynch Syndrome recommends offering low-dose aspirin (≤100 mg) for the prevention of CRC [7]. Ensuring chemoprevention is appropriately prescribed in the National Health Service (NHS) is listed as a priority within the Cancer Strategy for England [8]. A number of empirical studies and systematic reviews have highlighted a failure to translate research findings into changes in clinical care. A framework from Khoury and colleagues suggests 97% of genomic research involves developing successful clinical interventions for patients, with little focus on implementing successful developments [9]. The remaining 3% of research is directed towards evaluating the value of that intervention for clinical practice, the development and subsequent use of evidence-based guidelines by clinicians, and assessing the ‘real world’ impact of the intervention.

Attention is therefore required to ensure clinical recommendations developed following successful trial results are implemented rapidly. In addition, research should be undertaken to identify potential barriers to implementing future trial results, such as those from the CaPP3 study. Clinician-reported barriers to implementing preventive therapy guidelines for breast cancer have been reported [10–12], including the unlicensed status of tamoxifen, limited knowledge among general practitioners (GPs) and concerns about responsibility for prescribing.

As the use of aspirin for Lynch Syndrome carriers is still under investigation in clinical trials, it may be premature to investigate the role of licencing and care pathways as barriers to GP prescribing. However, knowledge and willingness to prescribe in this context are key factors that will affect subsequent implementation. Identifying such issues early will allow strategies to be developed to address them. In an Australian study (n = 181) of genetics professionals, gastroenterologists and colorectal surgeons, the majority (78%) reported having previously prescribed or recommended aspirin for Lynch Syndrome carriers [13]. However, the sample was small and unrepresentative, and GP attitudes were not investigated. Considering the majority of prescribing in the UK originates from general practice, this is an important group to include within attitudinal surveys in this context.

We surveyed a national sample of UK GPs currently practising in the NHS. GPs viewed relevant information on the topic and were asked a series of survey items investigating awareness of Lynch Syndrome and aspirin and their attitudes towards prescribing aspirin. We hypothesised GPs would be most willing to prescribe aspirin at lower doses. As 600 mg was the dose proven to reduce the risk of CRC associated with Lynch Syndrome in the CaPP2 trial [5], we investigated the GP characteristics associated with willingness to prescribe at this level. We anticipated a greater willingness to prescribe at the 600 mg dose among GPs who were more senior in their practice, had more experience as a GP, had prior knowledge of a cancer prevention indication for aspirin in Lynch Syndrome carriers, and reported a special interest in a relevant area. Finally, we aimed to report levels of awareness regarding Lynch Syndrome and the preventive effects of aspirin among carriers, as well as levels of comfort discussing the harms and benefits of aspirin with a Lynch Syndrome patient.

## Methods

### Sample

We conducted a national survey of GPs practising in the UK in April, 2016. Members of the M3 Global Research Panel, a private research company, were invited to take part in a survey via email. The M3 panel has over 33,000 GP members, which covers the vast majority of GPs in the UK. However, not all M3 panel members were approached to participate in the study. Panel membership is voluntary and respondents to surveys are paid for their time and expertise. In this instance, respondents were reimbursed £15 per GP.

Respondents were considered eligible if they listed general practice as their speciality. GPs practising outside of the UK were excluded. The survey company monitored recruitment rates to ensure all four UK nations were represented proportionately. A quota of 80 GPs from England with a role in clinical commissioning was targeted. Commissioners are responsible for planning, agreeing and monitoring services within the English NHS. University ethical approval was granted from the Queen Mary Ethics of Research Committee (QMREC1481).

### Study procedure and design

GPs were presented with brief information about Lynch Syndrome, including the approximate incidence of the syndrome in the population and the susceptibility of carriers to CRC and other cancers (online appendix). After items assessing GP’s awareness of LS, information was presented on the evidence for the cancer preventive effects of aspirin among this patient group. Specifically, GPs were presented with information regarding the outcomes of the CaPP2 trial [5], the recommended dose according to European guidelines [7], and the current status of the CaPP3 dose-inferiority trial. Respondents were informed of the major adverse events that can occur among people taking aspirin, and that aspirin was a generally accepted recommendation among this population group. Following this information, respondents completed the remaining items. GPs were able to consult the background information on Lynch Syndrome and aspirin throughout the items in this section.

The survey was co-designed by the authors of this report. Together, they have expertise in behavioural science, health policy, statistics, epidemiology, clinical genetics, primary care and public health. A draft survey was prepared by SS, and the remaining authors provided comments to be included in a revision. The survey was informed by a qualitative interview study investigating barriers to prescribing preventive therapy for breast cancer [10]. The interviews were done with family history and clinical genetics (FHCG) staff (n = 15) and general practitioners (n = 10). These data were supplemented by a further six interviews to develop the aspirin and Lynch Syndrome items.

### Study measures

#### Awareness of Lynch Syndrome and aspirin for cancer prevention

To assess knowledge of Lynch Syndrome, GPs were provided with some background information and asked, ‘Before today, had you heard of Lynch Syndrome, HNPCC or Muir Torre syndrome?’ Respondents were asked to indicate all that apply or ‘No, hadn’t heard of any’. GPs were also asked, ‘Have you ever seen a patient with Lynch Syndrome in your practice?’ (‘Yes’, ‘No’, ‘Unsure’).

To assess GPs’ awareness of the preventive effects of aspirin among this patient group, they were asked, ‘Before today, were you aware aspirin could reduce the risk of cancers associated with Lynch Syndrome?’ Response options were ‘Yes’ or ‘No’. Respondents were asked, ‘Have you ever discussed the use of aspirin with a Lynch Syndrome carrier’. Response options were, ‘Yes’, ‘No’ and ‘Unsure’.

#### Willingness to prescribe

To assess willingness to prescribe aspirin at each of the doses tested in the CaPP3 trial, GPs were asked to ‘Imagine the CaPP3 study shows that [100 mg/300 mg/600 mg] of aspirin is the optimal dose for reducing the incidence of cancer in Lynch Syndrome carriers. How willing would you be to prescribe aspirin [100 mg/300 mg/600 mg] for a patient with Lynch Syndrome?’ Response options were ‘Not at all willing’, ‘Probably not willing’, ‘Probably willing’ and ‘Definitely willing’. All respondents answered the question for each of the doses. The order in which the three doses were presented was randomised to prevent order effects. Data were combined to reflect unwilling and willing responses.

#### Comfort discussing harms and benefits

To assess GPs’ comfort in discussing aspirin with patients, respondents were asked, If a patient was recommended to take aspirin by a clinician in secondary care, how comfortable would you feel discussing the possible benefits and harms of aspirin with a Lynch Syndrome carrier? Response options were ‘very uncomfortable’, ‘quite uncomfortable’, ‘quite comfortable’ and ‘very comfortable’. Data were combined to reflect feeling comfortable and uncomfortable.

#### Respondent characteristics

GPs reported their gender, age in 10-year bands, status within the practice (‘GP specialist trainee’, ‘GP partner’, ‘Salaried or locum GP’, ‘GP retainer’ and ‘other’), region of practice (England, Scotland, Northern Ireland, Wales), year qualified in general practice (<10 years’ experience, ≥10 years’ experience), special interests (cancer, preventive medicine, family history, and genetics) and among those in England, their role in commissioning (yes, no).

### Statistical analyses

The trend for willingness to prescribe aspirin across the three doses was analysed using an extension of the Wilcoxon rank-sum test [14]. Multivariable logistic regression adjusting for all GP characteristics was used to test for group differences on awareness, comfort discussing the harms and benefits of aspirin, and willingness to prescribe at a 600 mg dose. Three multivariable models controlling for respondent characteristics tested the relationships between awareness of the preventive effects of aspirin for Lynch Syndrome, having seen a Lynch Syndrome patient in practice, and having heard of Lynch Syndrome with willingness to prescribe at 600 mg as the outcome. There were too few individuals reporting a GP status of ‘GP retainer’ or ‘other’ to be included in the logistic regression models, and therefore these individuals were excluded in these analyses. Statistical significance was set a priori at *p* < 0.05. Analyses were performed in SPSS version 22 and STATA version 12.

## Results

### Sample overview

In total, 13,764 GPs were emailed an invitation to take part with a link to the survey questionnaire, and 1321 started the survey (9.6% response rate). A total of 314 were excluded because they did not agree to the terms and conditions (n = 35), did not complete the survey (n = 143), completed the survey after the deadline (n = 35) or failed a data quality check undertaken by the survey company (n = 101). Data from 1007 GPs were available for analysis.

An overview of the study sample is shown in Table 1. In line with national data, the majority of GPs were from England (85.6%). Compared with national estimates, respondents were more likely to be salaried or locum GPs (38.5%), male (57.8%) and under the age of 50 years (72.3%). Over half of the respondents (56.2%) reported more than 10 years’ experience in general practice. A minority had a special interest in cancer (12.4%), preventive medicine (14.2%), family history (5.4%) and genetics (3.3%). Almost one-fifth (19.1%) of the English GPs reported having a role in commissioning.

**Table 1:** GP Sample and national characteristics (n = 1007)

	Sample (%)	National data (%) [15]
Country		
England	85.6	82.8
Scotland	7.8	9.8
Wales	3.9	4.7
Northern Ireland	2.7	2.7
Occupation		
GP partner	58.4	67.6
Salaried/locum GP	38.5	21.2
GP retainers	0.3	0.9
GP specialist trainee	2.0	10.3
Other	0.8	–
Gender		
Male	57.8	50.8
Female	42.2	49.2
Age		
<50	72.3	57.2
50+	27.7	38.0
Experience		
0–10 years	43.8	–
>10 years	56.2	–
Specialisms		
Cancer	12.4	–
Preventive medicine	14.2	–
Family history	5.4	–
Genetics	3.3	–

National data on experience and specialisms were unavailable

### Awareness of Lynch Syndrome and aspirin for cancer prevention

The majority of GPs (62.5%) reported they had not seen a patient with Lynch Syndrome in their practice, and 19.0% were unsure. Approximately one quarter (27.3%) of GPs had heard of Lynch Syndrome, and 61.2% had heard of HNPCC and 4.0% had heard of Muir-Torre Syndrome. Almost one-third (29.2%) of GPs had not heard of any of the names for the syndrome. In multivariable analysis adjusted for all GP characteristics, GPs were less likely to be aware of any names for Lynch Syndrome if they were male (OR 0.69, 95% CI 0.51–0.94, *p* = 0.021), working in Scotland (OR 0.53, 95% CI 0.31–0.88, *p* = 0.014) and 50 years or older (OR 0.42, 95% CI 0.30–0.58, *p* < 0.001).

Among GPs who had heard of Lynch Syndrome or any of its associated names, 3.8% reported they had discussed the use of aspirin with a Lynch Syndrome carrier. Among GPs who had heard of Lynch Syndrome or any of its associated names, only 46.7% were aware aspirin could reduce the risk of cancers associated with Lynch Syndrome. In a multivariable analysis restricted to those who were aware of Lynch Syndrome by at least one name, GP trainees had higher odds of being aware of the cancer preventive effects of aspirin than salaried/locum GPs (OR 3.55, 95% CI 1.00–12.62, *p* = 0.050). GPs older than 50 years of age had lower odds of being aware of the preventive effects of aspirin than their younger counterparts, although the effect was of borderline statistical significance (OR 0.66, 95% CI 0.43–1.01, *p* = 0.053).

### Attitudes towards discussing and prescribing aspirin

After reading information describing the preventive effects of aspirin among Lynch Syndrome carriers, 68.1% of all GPs indicated they would feel comfortable discussing the harms and benefits of the drug with a patient. There were no GP characteristics associated with reporting comfort in a multivariable model. GPs who were aware of the preventive effects of aspirin among Lynch Syndrome carriers were more likely to report feeling comfortable discussing the harms and benefits of aspirin (OR 1.81, 95% CI 1.36–2.43, *p* < 0.001).

**Fig. 1:**
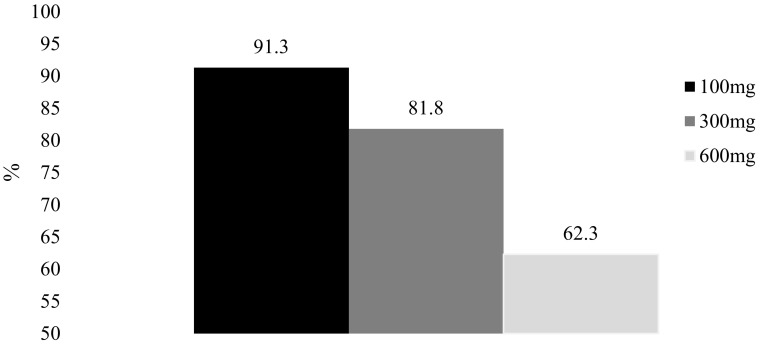
Willingness to prescribe aspirin at 100, 300 and 600 mg if the dose was shown to be optimal in the CaPP3 study (% willing) (N = 1007)

As shown in Fig. 1, almost all respondents indicated they were probably willing or definitely willing to prescribe aspirin at 100 mg (91.3%). There was a graded decline in willingness to prescribe aspirin at 300 mg (81.8%) and 600 mg (62.3%) (*p*-trend < 0.001).

In multivariable analysis, GPs who were older than 50 years of age were more likely to report a willingness to prescribe aspirin at 600 mg than their younger counterparts (OR 1.46, 95% CI 1.03–2.07, *p* = 0.033; Table 2). GPs with more than 10 years’ experience were also more likely to report a willingness to prescribe compared with less experienced GPs (OR 1.50, 95% CI 1.10–2.04, *p* = 0.010). In the opposite direction of our hypothesis, GPs reporting a special interest in family history were less willing to prescribe a 600 mg dose than those without this special interest (OR 0.41, 95% CI 0.21–0.82, *p* = 0.011). There were no differences in willingness to prescribe at the 600 mg dose among GPs with special interests in preventive medicine, cancer or genetics (*p* > 0.05).

**Table 2:** Willingness to prescribe aspirin (600 mg) for Lynch Syndrome by respondent characteristics (n = 1007)

	Unwilling	Willing	OR (95% CI)	*p*-value
Nation				
England	38.3	61.7	Ref	Ref
Scotland	29.1	70.9	1.54 (0.91–2.60)	0.108
Wales	46.2	53.8	0.69 (0.36–1.34)	0.277
Northern Ireland	33.3	66.7	1.22 (0.53–2.81)	0.639
GP Status (n = 996)				
GP partner	34.9	65.1	1.13 (0.85–1.50)	0.408
Salaried/locum GP	41.0	59.0	Ref	Ref
GP retainers	–	–	–	–
GP Specialist trainee	55.0	45.0	0.66 (0.26–1.65)	0.371
Other	–	–	–	–
Gender				
Male	35.7	64.3	1.14 (0.87–1.50)	0.339
Female	40.5	59.5	Ref	Ref
Age				
<50 years	41.3	58.7	Ref	Ref
50 years+	28.3	71.7	1.46 (1.03–2.07)	0.033
Experience				
0–10 years	45.8	54.2	Ref	Ref
>10 years	31.4	68.6	1.50 (1.10–2.04)	0.010
Cancer specialism				
Yes	40.0	60.0	1.02 (0.66–1.59)	0.919
No	37.4	62.6	Ref	Ref
Preventive medicine specialism				
Yes	37.1	62.9	1.23 (0.80–1.89)	0.348
No	37.8	62.2	Ref	Ref
Family history specialism				
Yes	51.9	48.1	0.41 (0.21–0.82)	0.011
No	36.9	63.1	Ref	Ref
Genetics specialism				
Yes	45.5	54.5	1.21 (0.53–2.77)	0.657
No	37.5	62.5	Ref	Ref

In further multivariable models controlling for all respondent characteristics, awareness of the preventive effects of aspirin among Lynch Syndrome carriers (OR 1.58, 95% CI 1.20–2.09, *p* = 0.001) and having seen a Lynch Syndrome patient in practice (OR 1.44, 95% CI 1.01–2.05, *p* = 0.045) were associated with a greater willingness to prescribe at the 600 mg dose. Reporting awareness of Lynch Syndrome or any of its alternative names was not associated with willingness to prescribe (OR 1.26, 95% CI 0.92–1.71, *p* = 0.151).

## Discussion

This national study highlights potential barriers to implementing the eventual findings of the CaPP3 trial in UK primary care. There was a statistically significant decline in willingness to prescribe aspirin for Lynch Syndrome carriers across the three doses being tested, with only two-thirds indicating they would prescribe at the 600 mg dose. Depending on the optimal dose identified by the CaPP3 trial, there could be important health-service barriers to implementing these research findings within routine clinical care. Furthermore, the highest quality evidence available from the CaPP2 study shows a dose of 600 mg is effective for cancer prevention in this population [5]. Lynch Syndrome patients seeking a 600 mg prescription of aspirin as part of their routine care may experience difficulties if seen by one of the significant minority of GPs who are reluctant to prescribe.

A barrier to ensuring Lynch Syndrome carriers receive appropriate care is likely to be low awareness among GPs regarding the syndrome and the preventive effects of aspirin among carriers. Almost one-third of GPs had not heard of any names of the syndrome, and HNPCC was the most commonly recognised name among those that had. This may be problematic because HNPCC does not account for cancers at multiple extracolonic sites that are integral to Lynch Syndrome. In our data, awareness of the syndrome did not necessarily translate to knowledge of the cancer preventive effects of aspirin among carriers. Importantly, reporting an awareness of the cancer preventive effects of aspirin and having seen a Lynch Syndrome patient in clinic were associated with a greater willingness to prescribe the 600 mg dose. While cross-sectional surveys do not allow causal inferences, it is possible that increasing awareness of preventive therapy in this context could facilitate prescribing behaviour. The National Institute for Health and Care Excellence (NICE), NHS England and the national equivalents may be best placed to take on such awareness-raising initiatives.

Educating GPs with regard to aspirin and Lynch Syndrome may be important for communicating with patients and promoting informed decision-making. Only two-thirds of GPs reported feeling comfortable discussing the harms and benefits of aspirin, and no sub-groups were identified as being particularly likely to report discomfort. There are currently no NICE national guidelines for the management of patients with Lynch Syndrome, which may be contributing to low awareness of the potential role of aspirin among Lynch Syndrome carriers. In support of the Cancer Strategy for England recommendation 7, we commend the decision by NICE to develop national guidelines for the management of Lynch Syndrome carriers [8]. In addition to guidance on screening and diagnosis, specific sections on the use of aspirin are needed. These guidelines should be updated following the results of the CaPP3 trial, which are expected in 2020.

Younger and less experienced GPs were less willing to prescribe aspirin in our data, yet were more likely to report an awareness of Lynch Syndrome and the cancer preventive effects of aspirin. This may suggest GPs graduating recently received more training in clinical genetics and preventive medicine. Alternatively, they may be better able to recall their training because it occurred more recently. Explanations notwithstanding, our observations suggest the effectiveness of awareness raising initiatives may be greater if they are targeted at older and more experienced GPs. Furthermore, our data also suggest alternative solutions to address prescribing behaviour may be needed for younger GPs who already demonstrate high awareness.

One possibility is to create a set care pathway for the prescription of aspirin. In a recent online vignette study we showed GPs are more willing to continue a tamoxifen prescription for breast cancer preventive therapy if a secondary care clinician has made the first prescription, compared with if the GP is asked to write the first prescription [11, 12]. A myriad of factors may be affecting this observation, but it suggests that changing health policy to implement shared care agreements between primary and secondary care may facilitate appropriate prescribing behaviour. In practice, this would mean prescriptions for aspirin would be initiated by clinical geneticists in secondary care and continued by GPs in primary care. However, clinical geneticists may be reluctant to prescribe at all [10], and genetic counsellors who often manage Lynch Syndrome patients do not have prescribing rights.

Anxiety regarding the lack of licence for preventive therapy medications more generally has been suggested as a major barrier to prescribing [11, 12]. Further research is needed to establish if this and other factors could affect willingness to prescribe aspirin for Lynch Syndrome. Attaining licences is unlikely, as there is no financial incentive for the pharmaceutical industry to undertake the work involved to achieve this. One strategy to overcome this barrier is to include cancer prevention as an indication for specific patient groups within the British National Formulary (BNF). The BNF is the primary resource used by UK prescribers when deciding on the appropriateness of a medication. While the BNF does not have the authority to licence a medication, it frequently describes alternative unlicensed indications for medications. The Access to Medical Treatments (Innovation) Act 2016 aimed at securing the repurposing of drugs for innovative purposes may have a role [15].

Targeting knowledge via guidance and education is necessary to improve the management of patients and potential drug side-effects. However, educating GPs is unlikely to be sufficient by itself to ensure population wide implementation of evidence-based practice. Assuming an average general practice list size of around 7200 patients, most practices will have between 6 and 18 people with Lynch Syndrome [16]. The majority of people with Lynch Syndrome are likely to be unrecognised [17], which is reflected in the low proportion of GPs who reported having seen a Lynch Syndrome patient in their practice. There are therefore further challenges for any strategy to change and habituate clinical practice for a relatively uncommon condition which is under-recognised and poorly recorded. A comprehensive population-level strategy therefore needs to include effective means to identify and code people with Lynch Syndrome. The newly launched patient support group, Lynch Syndrome UK, perhaps in conjunction with the more established cancer charities may be able to influence the low level of recognition of this treatable and preventable cancer category.

This study had limitations. The survey data were cross-sectional which prevents causal inference. Participants were recruited from a large online panel, but not all UK GPs are affiliated with the company responsible. Panel members may be more motivated to participate in research than non-members, and their survey responses may therefore not be generalisable to all primary care clinicians. Furthermore, only a small proportion of those who were approached agreed to take part in the survey. Attitudes and awareness among GPs who did not respond may therefore be different from those who participated. While the sample was representative with regard to country, respondents were more likely to be salaried GPs, male and younger compared with British Medical Association data [18]. This may further limit generalisability to those groups. Although the vignette was designed to mimic a clinical scenario as best as possible, the outcome data were hypothetical, and prescribing behaviour may be different within a clinical setting. A range of factors can affect decisions to prescribe, including knowledge differences in this context, and we could not account for all of them within this survey. For example, we did not provide exact information on the risk of bleeding associated with aspirin use in this population, or alternative risk reduction options. Further research is needed to understand the range of factors affecting clinician’s decision-making regarding aspirin prescribing for Lynch Syndrome carriers.

In conclusion, these data from a national sample of UK GPs highlight potentially important health-service barriers to implementing preventive therapy for Lynch Syndrome carriers, following the completion of the CaPP3 trial. Approximately one-third of GPs were unwilling to prescribe at the dose already demonstrated to be effective in the CaPP2 trial, and a third were uncomfortable discussing the harms and benefits of aspirin with a Lynch Syndrome carrier. Low awareness among GPs regarding Lynch Syndrome and the preventive effects of aspirin among carriers was also apparent. Developing national guidelines for the management of Lynch Syndrome carriers in tandem with campaigns to promote GP awareness and enhance case identification could overcome some barriers to prescribing and promote adequate communication on the topic. Adding cancer prevention as an indication for aspirin within the BNF may further reduce reluctance to prescribe.
